# Simulating 50 keV X-ray Photon Detection in Silicon with a Down-Conversion Layer

**DOI:** 10.3390/s21227566

**Published:** 2021-11-14

**Authors:** Kaitlin M. Anagnost, Eldred Lee, Zhehui Wang, Jifeng Liu, Eric R. Fossum

**Affiliations:** 1 Thayer School of Engineering at Dartmouth, Dartmouth College, Hanover, NH 03755, USA; Eldred.Lee.TH@dartmouth.edu (E.L.); Jifeng.Liu@dartmouth.edu (J.L.); Eric.R.Fossum@dartmouth.edu (E.R.F.); 2 Los Alamos National Laboratory, Los Alamos, NM 87545, USA; zwang@lanl.gov

**Keywords:** X-ray detection, direct detection, indirect detection, image sensor, scintillator

## Abstract

Simulation results are presented that explore an innovative, new design for X-ray detection in the 20–50 keV range that is an alternative to traditional direct and indirect detection methods. Typical indirect detection using a scintillator must trade-off between absorption efficiency and spatial resolution. With a high-Z layer that down-converts incident photons on top of a silicon detector, this design has increased absorption efficiency without sacrificing spatial resolution. Simulation results elucidate the relationship between the thickness of each layer and the number of photoelectrons generated. Further, the physics behind the production of electron-hole pairs in the silicon layer is studied via a second model to shed more light on the detector’s functionality. Together, the two models provide a greater understanding of this detector and reveal the potential of this novel form of X-ray detection.

## 1. Introduction

X-ray imaging with silicon and other semiconductors can be broadly divided into two categories. The first category is direct detection where the X-ray photon is absorbed directly in the silicon resulting in electron-hole pairs (EHPs) that are then collected and read out by the sensor. A second category is indirect detection where the X-ray photon is absorbed by a scintillating material layer and converted into many photons of lower energy, typically in the visible wavelength range. These photons then pass into a pixelated semiconductor detector where they generate EHPs like in a visible-light image sensor such as a charge-coupled device (CCD) or CMOS image sensor (CIS). Each category, direct and indirect, has associated advantages and disadvantages. 

**Figure 1:**
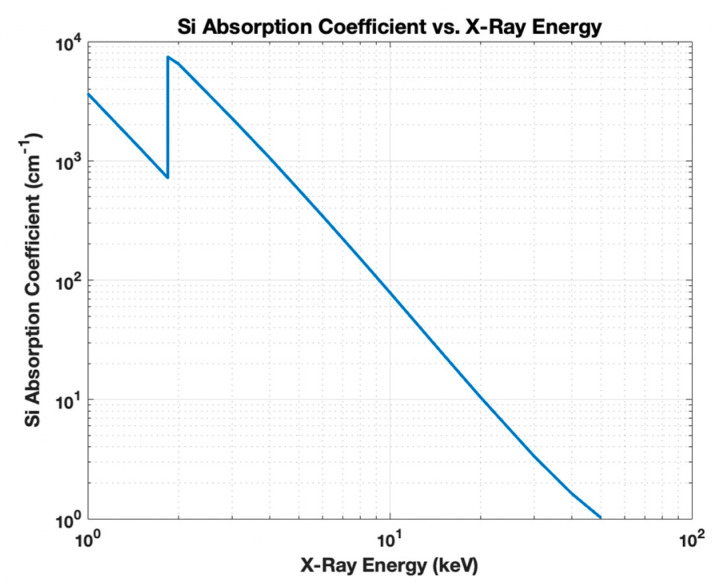
The absorption coefficient of Si vs. X-ray energy for 1–50 keV computed using the NIST database for Si [2].

In direct detection, the X-ray photon must be absorbed within the silicon, which becomes increasingly problematic at high energies due to low absorption coefficient. (see Figure 1—absorption coefficient vs. X-ray energy). At over 10 keV energies, the characteristic absorption length of greater than 100 μm makes it challenging to build efficient solid-state X-ray detectors and at 50 keV, only a very small fraction of the X-ray photon flux (~2%) can be absorbed by 200 μm thick silicon.

In indirect detection, the scintillator (e.g., CsI(Tl)) readily absorbs the X-ray and re-emits a number of lower energy photons [1]. However, the re-emission takes place over a large range of angles, so that for thicker scintillators, significant loss, crosstalk, and image “blurring” can occur when the re-emitted photons are absorbed in the underlying silicon pixels. Thin film scintillators, on the other hand, improve the spatial resolution at the cost of notably reduced quantum efficiency.

In this paper, an approach that is between direct and indirect detection is described and analyzed. This approach uses a Photon Attenuation Layer (PAL) made of high-Z material that inelastically scatters the high energy X-rays into lower energy X-rays. These lower energy X-rays then pass through the PAL to the semiconductor (Si) where their lower energy permits higher absorption rates and more efficient conversion of X-ray photons into EHPs and/or thinner detector thickness [3]. In the first part of the paper, this novel X-ray detector concept will be described, and the PAL modeled.

In performing modeling work of the PAL and semiconductor detector structure, we noted that a simple but complete qualitative description of a model of X-ray absorption to final EHP count (mean and “noise”) was not easy to find in the literature. Such a model is described in the second part of this paper to serve as a series of guideposts for future researchers, especially those that are more on the sensor and electronic device side.

Since the first medical X-ray image in America was taken at Dartmouth College in 1896 [4], X-ray imaging technology has become an integral part of imaging today. The two most common detector designs implement either direct detection, where the silicon detector is exposed to the radiation directly, or indirect detection, where a scintillator down-converts the X-rays to visible light before they strike the detector. The silicon must be very thick when using direct detection in order to sense higher-energy photons due to its low attenuation coefficient. However, a scintillator consisting of a luminescent, high-Z material may be placed over the detector to decrease the silicon’s required thickness. When a high-energy photon strikes the surface of a scintillator, it penetrates the material and generates lower-energy photons. The distance these particles spread increases with the depth of the material. If they scatter far enough, multiple pixels will detect them. This phenomenon, called charge-sharing, reduces the spatial resolution [1]. Thicker scintillators absorb a larger fraction of the incident radiation than thinner ones—they have higher absorption efficiency—but at the cost of more charge-sharing [5]. To combat these shortcomings, an innovative design was proposed in [3], consisting of a photon-attenuation layer (PAL) above a silicon detector. The proximity of the PAL to the silicon layer may yield enhanced absorption efficiency without or significantly mitigate additional charge sharing. The first purpose of the paper is to describe this novel device structure and modeling results. However, to design a prototype, it is also necessary to know the number of electron-hole pairs generated and collected in the silicon after an X-ray strikes it. While the average number of carriers generated by an X-ray photon of energy E (eV) is well-known to be E/W, where W is the pair-creation energy and W = 3.65 eV for silicon [6], understanding the standard deviation (noise) in this quantity has not been well explored beyond the standard model. According to the standard model, the fluctuation of EHPs is proportional to (fE/W)^1/2^, with f, called the Fano factor, is about 0.13 for silicon [7]. Further, a simple but complete end-to-end description of the modeling process seems to be missing from the literature. Thus, another purpose of this paper is to provide a description of the modeling process that may be useful to other sensor designers. This process includes a Monte Carlo (MC) simulation to clarify the physics behind the system and determine the number of electron-hole pairs produced and the standard deviation, the Fano factor, and the pair-creation energy as a result of an incident X-ray on the system.

## 2. PAL-Si Simulations

**Figure 2:**
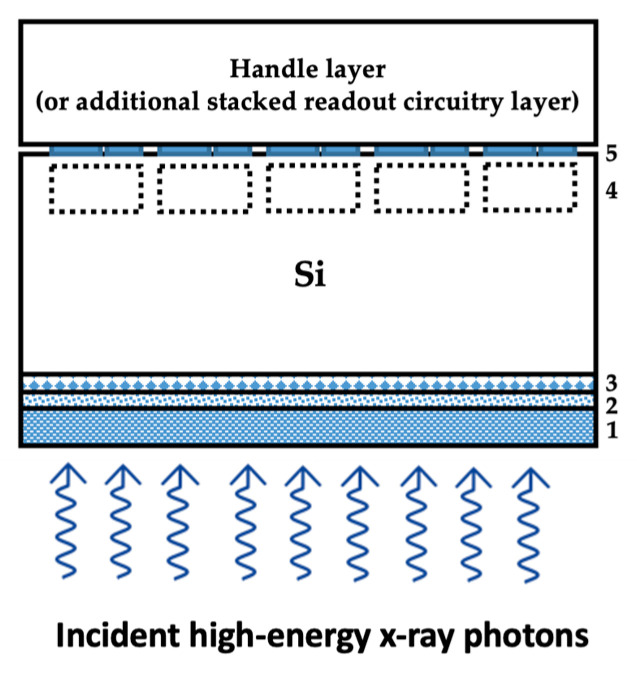
A schematic of the cross-section of the PAL-Si detector system. Region 1 is the PAL, 2 is a backside passivation oxide layer, 3 is either implants or epitaxial growth for surface pinning, region 4 is the pixelated carrier storage wells, and 5 is front side pixel readout circuitry or hybrid bonds to 3D stacked readout circuitry.

The system is comprised of the lead telluride (PbTe) PAL on a silicon (Si) detector such as a CMOS Image Sensor (CIS). If the detector is backside illuminated (BSI), as shown in Figure 2, the PAL is deposited below the Si so the X-rays strike the PbTe layer first. The PAL is placed on top of the Si in a frontside illuminated (FSI) detector. When an X-ray with energy 20–50 keV strikes the PAL, it is down-converted such that most photons have less energy than the incidental X-ray. However, most photons remain in the X-ray regime and are not converted to visible or ultraviolet light, unlike typical indirect detection methods using scintillators. The PAL therefore does not need to be as thick because the X-rays do not lose as much energy, resulting in improved spatial resolution. At this lower energy level, the photons are much more likely to be absorbed in the detector to generate EHPs for readout compared to the direct detection method. The PAL serves as a method to minimize the thickness of the Si, thereby reducing the trade-off between absorption efficiency and spatial resolution. With thinner Si, the detector can have improved characteristics including higher spatial resolution, faster readout, and more. Like conventional scintillators, the PAL re-emits X-ray photons that are scattered into a large solid angle, a consideration when designing the aspect ratio of pixel pitch to depth. Larger aspect ratio reduces crosstalk but greater Si depth improves the likelihood of absorption. Unlike scintillators, the PAL interaction volume is much smaller, likely allowing a better trade-off for detector resolution and quantum efficiency.

The Si and PAL thicknesses are chosen to account for the attenuation coefficients of the incident X-rays. Higher energy particles require thicker PbTe and Si to be efficiently down-converted and detected, while lower-energy particles would be shielded with thick PbTe and recombine in thick Si. In order to quantify the relationship between the PAL and Si thicknesses and the number of electrons generated, simulations in Monte Carlo N-Particle Software (MCNP) 6.2 [8] were conducted in which 100,000 photons with energy from 20 to 50 keV strike the PAL and Si a single time. The PAL and Si layer ranged in thickness from 0.1 to 5 μm and 5 to 200 μm, respectively. Due to the 20–50 keV incident photon energy and higher-Z PAL absorber, the photoelectric effect is the main absorption mechanism in this simulation. The photoelectric effect has increasing dominance with increasing Z-number and decreasing energy, but Compton scattering does occur in small quantities [9]. Consequently, Compton scattering was included in the simulation, but had a minimal effect on the results. Additionally, the model considers down-converted X-ray photons that are reabsorbed in the PAL, resulting in fewer photons striking the Si detector.

**Figure 3:**
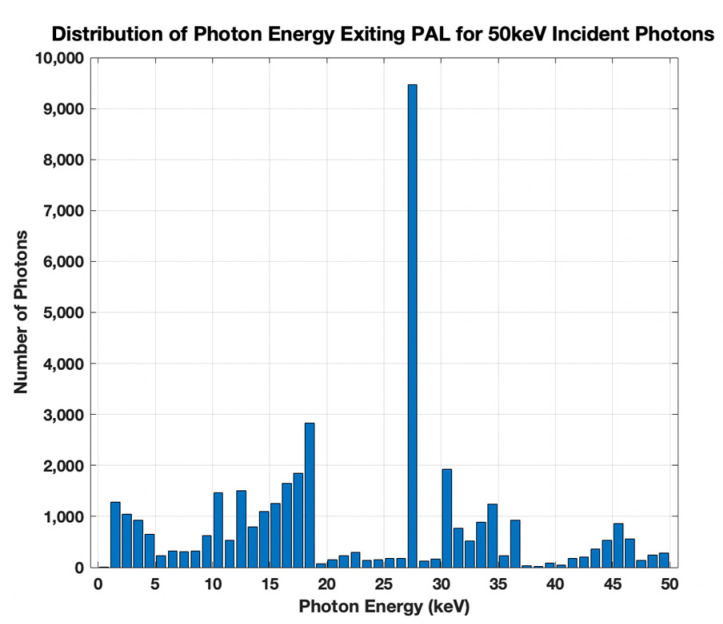
The energy distribution of the photons exiting the 0.5-μm thick PAL and entering the Si detector for 100,000 50-keV incident X-ray photons. The most prominent line is at Te KL_2_ (27.2 keV) and Te KL_3_ (27.5 keV). Te KM_2_ (30.9 keV) and Te KM_3_ (31.0 keV) are also visible.

The simulation outputs the number of photons exiting the PAL in ΔE = 1 keV bins: 0–1 keV, 1–2 keV, etc. One example is illustrated in Figure 3 for 50 keV incident photons and 0.5-μm thick PAL. The distinct peaks are attributed to be either characteristic X-rays or shell edges from Pb and Te. In particular, the most prominent peak at 27.5 keV corresponds to the KL_2_–KL_3_ transition for Te [2]. These down-converted photons will then enter the Si detector and either be absorbed or transmitted. The density of normal-incidence photons of energy E, P(L,Ε) absorbed in the Si layer of thickness L, can be calculated using Beer–Lambert’s law, [10]:

(1) $$P\left(L,E\right){=P}_{\mathrm{in}}\left(E\right)\left[1-e^{-\mu (E)L}\right],$$

where P_in_(Ε) is the photon density impinging the Si and μ(E) is the linear attenuation coefficient. The linear attenuation coefficients were found using the mass attenuation coefficients from [11].

Next, the number of photoelectrons generated N_gen_ from Si over the energy range E_min_ to E_max_ is given by the integration of (1):

(2) $$N_{\mathrm{gen}}=\int_{E_{\min}}^{E_{\max}}\frac{P(L,E)\cdot E}{W}\mathrm{dE},$$

This expression is approximated in simulation by:

(3) $$N_{\mathrm{gen}}\;\approx \sum_{m=1}^{E_{\max}/\Delta E\;}\frac{{P(L,E}_{m})\cdot E_{m}}{W}\Delta E,$$

where the bin number is m, the average energy in bin m is E_m_ = $\left(m-\frac{1}{2}\right)\;\Delta E$, and ΔE=1 keV in the calculations. As mentioned above, W = 0.00365 keV is the mean amount of energy required to generate one electron-hole pair [6], which assumes all absorbed X-ray photons convert entirely to EHPs. While the simulation produces fluoresced and scattered photons over a range of 4π steradians, it does not report the exact number that are orthogonal. Therefore, half of all photons exiting the PAL are assumed to be absorbed in the detector to account for those that are scattered and fluoresced at large angles.

**Figure 4:**
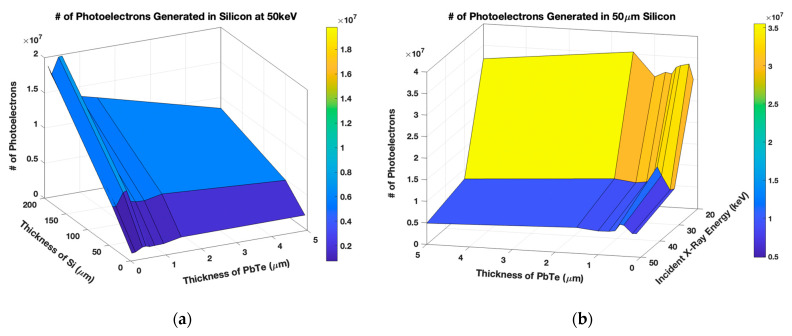
(**a**) The number of photoelectrons generated with 50 keV incident photons, 5–200 μm Si, and 0.1–5 μm PbTe. (**b**) The simulation using 20–50 keV photons, 50 μm Si, and 0.1–5 μm PbTe.

Figure 4a shows the number of photoelectrons generated using 5–200 μm thick Si, 0.1–5 μm thick PbTe, and 50-keV incident X-rays. The number of photoelectrons grows with increasing Si thickness as predicted because more photons are absorbed. The optimal PbTe thickness appears to be at 0.5 μm PbTe where the PAL down-converts the photons, but doesn’t shield the Si. Thicker PbTe blocks the down-converted photons from the Si, yielding fewer photoelectrons.

The simulation was also performed using 50 μm thick Si, 0.1–5 μm thick PbTe, and 20–50 keV incident X-rays, illustrated in Figure 4b. The number of photoelectrons generated decreases with increasing incident photon energy since higher-energy photons are not down-converted as efficiently by the PAL. Despite the optimal thickness of PbTe occurring at 0.5 μm in the limited number of simulation runs, as indicated by the fold in the Figure, the number of pairs does not decrease as expected. Instead, there is a dip at 1 μm PbTe before increasing at 1.5 μm PbTe to be comparable to the 0.5 μm results. The 0.5 μm PbTe yields more higher-energy photons while the 1.5 μm thick PbTe produces more lower-energy photons that generate a similar number of photoelectrons. The 1 μm PbTe in turn yields fewer higher-energy photons than 0.5 μm PbTe and fewer lower-energy photons than the 1 μm PbTe, resulting in the decline.

Additionally, the number of photoelectrons generated before impact ionization divided by the number of incident photons, called the quantum yield (QY), is a useful parameter to compare to existing technologies. The QY of 1 μm PAL, 20 keV X-rays, and 50 μm Si is ~20%, much greater than the <5% using the silicon direct detection method [3,12]. A similar technology using a photocathode only obtained 5% photoelectron generation with 7.5-keV photons [13]. As previously mentioned, MCNP does not specify the number of photons exiting the PAL orthogonally, which may lead to an overestimation of the QY. On the other hand, MCNP’s default cutoff photon energy value is below roughly 1 keV, which may underestimate the QY [3]. However, recent experimental results awaiting publication demonstrate significant relative signal enhancement from the Si reference. The PAL concept’s QY, while an estimation, appears to be much greater than competing detection methods overall.

## 3. Monte Carlo Model

### 3.1. Relaxation and Cascade Process

After the down-converted photons exit the PAL, they enter the Si detector and generate photoelectrons. While many models were developed to determine the average energy required to generate an EHP in Si, the associated comprehensive qualitative discussion is more difficult to find. The MC model discussed in this section was developed to clarify the process of photons striking a Si detector and generating EHPs to better understand the PAL concept.

First, an X-ray photon is simulated striking the Si detector and producing electron-hole pairs via non-radiative transitions like photoelectric absorption and Auger emissions [14]. In an Auger process, the inner shell electron removed from the atom by photoionization, called a photoelectron, induces an outer shell electron to drop to the inner shell, thereby releasing a second electron called an Auger electron [15]. The two vacancies that remain and the electron that was released are collectively known as primary carriers. The probability of the incident photon being absorbed into particular shells is energy dependent up to 1839 eV; after this, there is a constant 92% and 8% probability of K-shell and L_1_-shell absorption, respectively [16,17,18]. In this model, only photons with 5-keV energy and above were simulated.

After vacancies are created in an atom’s inner shells, these holes will shift to the atom’s outer shells via Auger decays as part of a vacancy cascade. The probabilities for the possible Auger processes and values of the Auger electrons used in the model are those listed in Figure 2 of [17]. The convention for assigning energy values for the resulting holes is that used in Section 3 of [18]. The vacancy cascade continues until all holes are pushed into the valence band edge at the M_1_ or 3s shell in Si.

During the vacancy cascade and photoionization process, core electrons being removed may give bound valence electrons some of their kinetic energy while passing through the valence band, thereby exciting them. These bound electrons may release some of their excess energy by either moving to a higher energy band within the atom (electron shake-up) or by being ejected from it entirely (electron shake-off) [19]. This model neglects the possibility of electron shake-up, like others [20] because if a valence electron becomes excited enough to jump to a higher state, it also likely has sufficient energy to be emitted from the atom completely [21]. The probabilities of electron shake-off, values of the shaken-off electrons, and the relaxation energies used here are shown in Table 1. The absolute probability of shake-off occurring as a result of a vacancy in a particular shell and values of the shaken-off electrons were found by averaging the values from Tables I and II from [22] for neon and argon, similar to [17]. For simplicity, the total probability of a shake occurring as a result of a vacancy in a particular shell was used. The amount of energy required to cause electron shake-off is called relaxation energy (E_r_). The E_r_ values used were those for Si in Table III in [19] for 1s, 2s, and 2p shells; the 3s shell value was obtained by averaging aluminum’s and sulfur’s since Si’s was not listed.

To account for the energy loss from shakes, the convention used was as follows:

(4) $$E_{\mathrm{new}}{=E}_{0}\;{-\;E}_{r}\;{-\;E}_{\mathrm{se}},$$

where E_new_ is the new energy of the primary carrier or photoelectron that caused the shake-off, E_0_ is its energy pre-shake-off, and E_se_ is the energy of the shaken-off electron. In an Auger decay, it is assumed that E_r_ and E_se_ are subtracted from the photoelectron when the shake results from the first or second shell. When the shake results from the third shell, E_r_ and E_se_ are subtracted from the primary photoelectron. Since some of the transitions in [17] do not specify which M-shell was involved, these shells are presumed to be deeper in the valence shell; thus, the probability of shake-off is neglected. The energy of the holes resulting from shake-offs is, for simplicity, assumed to be uniformly distributed in energy across the 12-eV width of the valence band [23]. The vacancy cascade continues until all holes except those generated from shakes move to the valence band edge or deeper. The probability of shake-off occurring in Table 1 does not specify the shell from which the electron is shaken-off, so these holes cannot be shifted to the outer edge in the simulation.

The photoelectrons, primary carriers, shaken-off electrons, and the resulting holes generated from the vacancy cascade next take part in a cascade process in which they shed their excess energy. A visualization of the cascade may be found in Figure 2 of [24]. This step ends when all carriers are no longer excited and have less than the threshold energy E_th_ = 3/2E_gap_, where E_gap_ is the bandgap of Si, obeying the conservation of energy and momentum [25]. When an electron or hole releases its extra energy, it can scatter by either phonon emission—giving up energy in the form of lattice vibrations [26], or ionization—creating an electron-hole pair. Both carriers created via ionization, called secondary carriers, have the capability of continuing the cascade processes if they have energies greater than or equal to E_th_. If the primary carrier has an original energy of E, one secondary carrier it produces has an energy of E_1_ = $\alpha_{1}$(E − E_gap_), while the other secondary carrier has an energy of E_2_ = $\alpha_{2}$(E − E_gap_ − E_1_) [18]. The coefficients $\alpha_{1}$ and $\alpha_{2}$ are random numbers between 0 and 1 that can be found by solving (7) and (10) in [27]:

(5) $$R_{1}=\frac{105}{8}\alpha_{1}^{3/2}\left[\frac{\alpha_{1}^{2}}{7}-\frac{{2\alpha }_{1}}{5}+\frac{1}{3}\right]$$

(6) R2=(2π)(2α2-1)α2(1-α2)12+π-1sin-1(2α2-1)+12 

R_1_ and R_2_ are uniformly distributed random numbers between 0 and 1. In order to improve simulation time, these equations are solved for $\alpha_{1}$ and $\alpha_{2}$ in advance and the results are stored in a matrix of over 4 million values that is shuffled at the start of each cascade. After plugging in said values to solve for E_1_ and E_2_, the energy of the primary carrier post-scattering then becomes E_new_ = E − (E_1_ + E_2_ + E_gap_). The probability the primary carrier will scatter via ionization P_0_(E) is determined from (7) in [25], given as:

(7) $$P_{0}(E)=\frac{1}{1+{\left(r^{\prime }(E)/r(E)\right)}^{\prime }}$$

where r′(E) is the rate of scattering via phonon emission for a particle of energy E and r(E) is that particle’s rate of scattering via ionization. The ratio of these two variables is given in (20) from [25]:

(8) $$\frac{r^{\prime }(E)}{r(E)}=A\frac{105}{2\pi }\frac{{\left(E-\hbar \omega_{0}\right)}^{1/2}}{{\left(E-E_{\mathrm{gap}}\right)}^{7/2}}$$

The constant A is chosen to be 5.2 eV^3^ [25] (p. 5574), the energy of an optical phonon $\hbar \omega_{0}$ in Si is 63 meV, and E is the energy of the carrier being cascaded [25]. The temperature is assumed to be 300 K, yielding a value of 1.12 eV for E_gap_ via the Varshni equation [26]. At the beginning of each cascade, the carrier is assigned a uniformly distributed random number between 0 and 1. If this number is less than P_0_(E), the carrier will scatter by ionization, and if not, it will scatter via phonon emission. This model uses the free particle model and the “scattering rate assumption” developed in [25] and employed in [18]. Additionally, the Si is assumed to be wide and thick enough for the cascade to continue to completion. Energy loss via acoustic phonon emission is neglected and only optical phonon emission is considered. However, a recent study [28] suggests acoustic phonon emission may be a dominant energy loss mechanism and may be considered in future versions of this model.

### 3.2. Results

**Figure 5:**
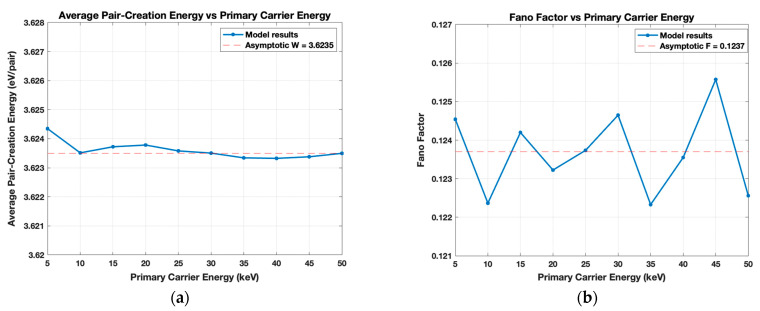
Cascade results as a function of 5–50 keV primary carrier energy. (**a**) The average pair-creation energy with asymptotic value ~3.63 eV/pair; (**b**) Fano Factor that fluctuates at about ~0.124.

The cascade process was modeled 10,000 times with a single primary carrier at varying energy levels between 5 and 50 keV. Figure 5a shows the amount of energy required to generate an electron-hole pair, or the pair-creation energy *W*, as a function of primary carrier energy. It was calculated by dividing the primary carrier’s initial energy by the average number of secondary pairs generated in each trial $\mu_{n_{p}}$, or:

(9) $$\mu_{n_{p}}=\frac{n_{p}}{N},$$

where n_p_ is the total number of pairs generated in all N trials:

(10) $$n_{p}=\overset{N\;}{\underset{i=1}{\sum }}{\left(\#\;\mathrm{of}\;\mathrm{pairs}\;\mathrm{generated}\right)}_{i}\;$$

*W* is mostly constant at ~3.63 eV/pair with a slight decrease as energy increases, which is consistent with literature [23,26,27]. The fluctuation in *W* may also be of interest and can be evaluated with the Fano factor *F*. The equation for F, given by (3) in [29], is:

(11) $$F=\frac{\sigma_{n_{p}}{}^{2}}{\mu_{n_{p}}},$$

where $\sigma_{n_{p}}{}^{2}$ is the variance of the number of pairs generated in each trial:

(12) $$\sigma_{n_{p}}{}^{2}=\frac{n^{2}{}_{p}}{N}{-\mu }_{n_{p}}{}^{2},$$

and $n^{2}{}_{p}$ is the sum of the square of the number of pairs generated in each run. The Fano factor for each energy level was computed using this method and is plotted in Figure 5b. *F* fluctuates due to the statistical nature of the process, but the asymptotic value is ~0.124. Both the trend and asymptotic value in Figure 5b agree with literature [25].

**Figure 6:**
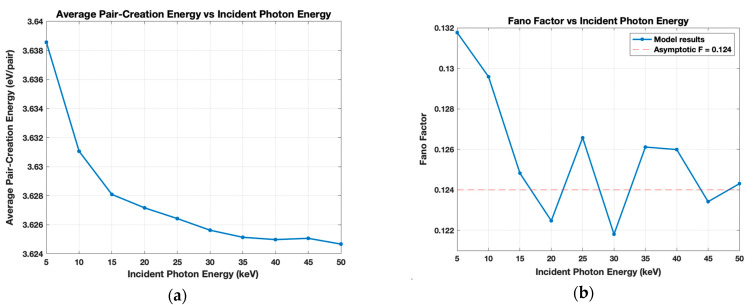
Full model results for 5–50-keV incident photon energy. (**a**) The average pair-creation energy that tends to ~3.63 eV/pair; (**b**) Fano Factor with an asymptotic value 0.124.

The full MC simulation in which a single photon with varying incident energy levels strikes Si was also performed 10,000 times to determine *W* and *F* in the same 5–50 keV energy range. These results are shown in Figure 6a,b, respectively, which exhibit similar results to those produced by the cascade simulation. The presence of the vacancy cascade in the full model causes the steep decrease in W with increasing energy. In this process, relaxation energy is lost by the primary carriers and in turn causes fewer secondary carriers to be produced, leading to larger W values. This effect is minimal at higher incident photon energies, but may be more substantial at lower energies, leading to the trend in Figure 6a. In Figure 6b, the asymptotic value of F, 0.124, is nearly identical to the 0.1237 value obtained by the cascade. The decreasing value of F with increasing energy is reasonably consistent with Gao et al. in [30], but they report F to tend towards 0.135. This slight discrepancy may be attributable to the pre-determined matrix of $\alpha_{1}$ and $\alpha_{2}$ values used in the cascade portion of the simulation. If the number of calculations is great enough, the $\alpha_{1}$ and $\alpha_{2}$ values are reused multiple times in the same MC simulation. There will then be less variation in the data, yielding a smaller F. Gao et al. [30] also state the use of the 1970 Cromer and Liberman database—used here in this model—may lead to inaccuracies in the results around the shell edges due to being an outdated reference. However, this MC simulation only includes energy values greater than these edges and the results above the K-edge appear to be similar to those in other references [23,26,31].

**Figure 7:**
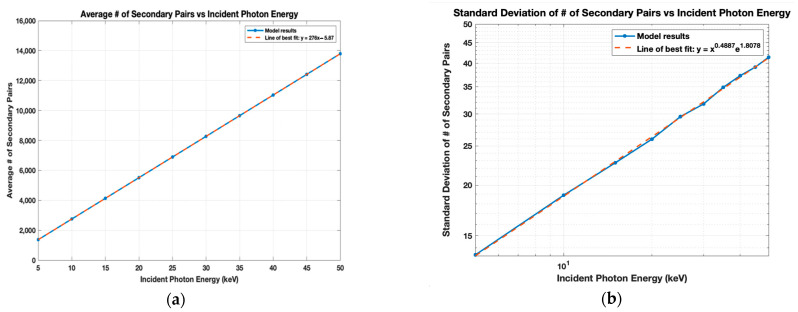
Full model results. (**a**) The average number of secondary pairs created; (**b**) The standard deviation of the number of pairs. The best fit and model values have a standard deviation of less than 1%.

In addition to the W and F values, the distribution of $\mu_{n_{p}}$ and its corresponding standard deviation σ were found. $\mu_{n_{p}}$ is plotted in Figure 7a for 5–50 keV and depicts a linear relationship between $\mu_{n_{p}}$ and the incident photon’s energy, with the line of best fit being y = 276x − 5.87. Although not shown, a Gaussian fit was applied to the data at each energy level and the σ found were very consistent with the distribution for the cascade. Despite the $\mu_{n_{p}}$ distribution appearing to be Gaussian, Fraser et al. noted the asymmetry of the Gaussian peaks at lower energy levels, so σ was instead computed by rearranging the equation for *F*: $\sigma =\sqrt{\mu_{n_{p}}\times \;F}$. The model’s logarithmic results for σ in Figure 7b were fitted to a linear regression with the equation ${y=x}^{0.4887}e^{1.8078}$. The trend lines obtained for the full model were nearly identical to those for the cascade model; therefore, only the full model results are illustrated. Both trend lines may be used to predict $\mu_{n_{p}}$ and σ at particular energy levels.

The average charge of the Si ion post-vacancy cascade is roughly +5, in agreement with Fraser et al., but the ‘recipe’ developed in [32] predicts a value of +4. This disagreement may be due to the exclusion of fluorescence, which creates only one vacancy, while an Auger process generates two. This difference may grow in the vacancy cascade as each hole is moved to the outer shell and initiates more Auger decays. Given the 4.4% probability of fluorescence occurring after a K-shell photon absorption [17], it is likely over 10,000 trials that this phenomenon is statistically significant and would cause this discrepancy. Nevertheless, fluorescence was neglected because $\mu_{n_{p}}$ would be much lower and cause F to diverge unless the emitted photon was reabsorbed in the Si.

## 4. Conclusions

This paper couples two models together to clarify the physics behind a new, promising form of high-energy X-ray detection. Simulations using MCNP elucidate the properties of the PAL while the MC model clarifies the physics of the electron generation in the Si detector. The MCNP simulation results confirm the authors’ predictions regarding thicker PAL shielding the Si from incident X-rays and thinner PAL down-converting few X-rays. The data indicate that 0.5 μm PbTe is the optimal thickness for a 50 μm thick Si detector, which may be used to fabricate a future prototype. Additionally, the MC model provides a start to finish qualitative and quantitative description of photons striking the Si layer and generating EHPs, which may be useful to other sensor designers. The results validate the approximation of ~3.65 eV/pair frequently used in literature. The trend lines found for $\mu_{n_{p}}$ and σ may also be helpful to others. This model paired with MCNP paints a comprehensive picture of how the PAL concept will function and gives insight into its potential advantages over traditional indirect and direct detection solutions used today. Test chips for the prototype were fabricated and tested pre- and post-deposition. Due to material unavailability and equipment contamination concerns, Bi_2_Te_3_ was used instead of PbTe. The relative signal enhancement post-deposition was significantly larger than that of pre-deposition [33]. This experimental data may be compared with the theoretical results presented here in a future publication. While the data reported in this paper are theoretical, they demonstrate the physics of this alternative X-ray detection method that may expand the limits of this field.

## Figures and Tables

**Table 1:** The shell in which a vacancy caused a shake, the absolute probability of a shake occurring, the value of the shaken-off electron, and the relaxation energy associated with that shake-off.

Shell	Absolute Probability of Shake-Off (%)	Energy Value of Shaken-Off Electron (eV)	Relaxation Energy E_r_ (eV)
K (1s)	19.75	19.20	27.1
L_1_ (2s)	9.45	6.25	7.0
L_2,3_ (2p)	9.70	6.55	8.0
M_1_ (3s)	9.65	6.55	1.2

## Data Availability

The data presented in this study are available on request from the corresponding author.
